# The effect of hyperglycemia on neurovascular coupling and cerebrovascular patterning in zebrafish

**DOI:** 10.1177/0271678X18810615

**Published:** 2018-11-06

**Authors:** Karishma Chhabria, Karen Plant, Oliver Bandmann, Robert N Wilkinson, Chris Martin, Elisabeth Kugler, Paul A Armitage, Paola LM Santoscoy, Vincent T Cunliffe, Jan Huisken, Alexander McGown, Tennore Ramesh, Tim JA Chico, Clare Howarth

**Affiliations:** 1 Neuroimaging in Cardiovascular Disease (NICAD) Network, University of Sheffield, Sheffield, UK; 2 Department of Infection, Immunity and Cardiovascular Disease, University of Sheffield Medical School, Sheffield, UK; 3 The Bateson Centre, University of Sheffield, Sheffield, UK; 4 Department of Neuroscience, University of Sheffield Medical School, Sheffield, UK; 5 Department of Psychology, University of Sheffield, Sheffield, UK; 6 Department of Biomedical Science, University of Sheffield, Sheffield, UK; 7 Morgridge Institute for Research, Madison, WI, USA

**Keywords:** Cerebrovascular patterning, diabetes, neurovascular coupling, nitric oxide, zebrafish

## Abstract

Neurovascular coupling (through which local cerebral blood flow changes in response to neural activation are mediated) is impaired in many diseases including diabetes. Current preclinical rodent models of neurovascular coupling rely on invasive surgery and instrumentation, but transgenic zebrafish coupled with advances in imaging techniques allow non-invasive quantification of cerebrovascular anatomy, neural activation, and cerebral vessel haemodynamics. We therefore established a novel non-invasive, non-anaesthetised zebrafish larval model of neurovascular coupling, in which visual stimulus evokes neuronal activation in the optic tectum that is associated with a specific increase in red blood cell speed in tectal blood vessels. We applied this model to the examination of the effect of glucose exposure on cerebrovascular patterning and neurovascular coupling. We found that chronic exposure of zebrafish to glucose impaired tectal blood vessel patterning and neurovascular coupling. The nitric oxide donor sodium nitroprusside rescued all these adverse effects of glucose exposure on cerebrovascular patterning and function. Our results establish the first non-mammalian model of neurovascular coupling, offering the potential to perform more rapid genetic modifications and high-throughput screening than is currently possible using rodents. Furthermore, using this zebrafish model, we reveal a potential strategy to ameliorate the effects of hyperglycemia on cerebrovascular function.

## Introduction

Neurovascular coupling refers to the mechanism by which neural activation evokes a local increase in cerebral blood flow.^1,2^ Impaired neurovascular coupling is proposed to underlie a range of disorders including neurodegeneration and dementia^3–5^ and is impaired in animal models of diabetes.^4,6^ Preclinical studies of neurovascular coupling currently rely on mammalian models, which are highly invasive, requiring surgical exposure and instrumentation of the brain. A less invasive model would therefore be desirable. The zebrafish is an established model for studying the development of the brain^7,8^ and vasculature^9^ and is increasingly used to model human disease.^10^ Advanced imaging, particularly single plane illumination microscopy (SPIM)^11^ provides rapid imaging allowing non-invasive observation of dynamic phenomena such as neuronal activation or blood flow in living animals. Drugs can be administered by immersion, allowing rapid testing of potentially therapeutic compounds.^12^ These advantages allow novel approaches to understanding mechanisms of disease and identifying potential therapeutics.

We here describe non-invasive quantification of neurovascular anatomy, haemodynamics and neural activation simultaneously in non-anaesthetised zebrafish. We show that, like mammals, zebrafish larvae after a certain stage of development exhibit neurovascular coupling. Chronic exposure of larvae to glucose impairs both cerebrovascular patterning and neurovascular coupling, and reduced expression of a reporter expressed in the blood–brain barrier. Lastly, sodium nitroprusside (SNP), a drug widely used clinically for hypertension^13^ was able to reverse all the adverse effects of hyperglycaemia on cerebrovascular development and neurovascular coupling, uncovering a possible therapeutic strategy to protect the brain from the effects of diabetes.

## Methods

### Transgenic zebrafish

Zebrafish studies were in accordance with the Animals (Scientific Procedures) Act, 1986, United Kingdom and were performed under Home Office Project licence 70/8588 held by TC. All procedures were approved by the Ethical Review Committee at the University of Sheffield. Reporting of these experiments complies with the ARRIVE (Animal Research: Reporting in Vivo Experiments) guidelines. Zebrafish expressed three transgenes: *kdrl:mCherry*,^14^ *gata1:DsRed*^15^ and *nbt:GCaMP3* expressing the genetically encoded calcium reporter GCaMP3 under control of the neuronal beta-tubulin (NBT) promoter.^16^ For the *claudin5a:GFP* reporter expression studies, zebrafish expressed the transgenes *claudin5a:GFP*^17^ and *kdrl:mCherry*.^14^

Adult fish were maintained on a 14/10-h light/dark cycle and tanks of transgenic carriers incrossed to generate embryos for the studies described. These were raised according to standard protocols.^18^

### Lightsheet microscopy

Non-anaesthetised zebrafish at the indicated age were mounted individually in 3% low melting point (LMP) agarose (Sigma) within a 1 mm diameter glass capillary bathed in E3 medium (5 mM NaCl, 0.17 mM KCl, 0.33 mM CaCl_2_, 0.33 mM MgSO_4_) within the chamber of a Zeiss Z1 lightsheet microscope maintained at 28 ℃. The region of interest (ROI) was excited with a lightsheet generated by 488 nm (50 mW) and 561 nm (50 mW) lasers sequentially; 488 nm laser power was 30–50% and 561 nm laser power 1–2%. Emitted light was collected using an LP560 filter.

To visualise cerebrovascular anatomy in the optic tectum, a 90–100 µm Z stack was obtained with 1 µm slices.

To visualise neurovascular coupling, the animal was left for 3–4 min in the dark to acclimatise before a single sagittal Z slice was imaged serially at 32 frames/s at the level of the superior granular layer of the optic tectum (stratum opticum). After 29 s baseline imaging, a 5 V red (700 nm) light emitting diode (LED) inside the chamber was used to administer a visual stimulus lasting 8 s. After the stimulus ended, imaging continued for a further 29 s. The total time series (66 s) dataset therefore comprised 2100 frames. This was performed twice per animal.

### Glucose, mannitol, and SNP exposure

Glucose, mannitol, and SNP (Sigma) were dissolved in E3 medium to final concentrations of 20 mM (360 mg/dL) (glucose and mannitol) or 0.1 mM (SNP).

Embryos were incubated in E3 medium containing these drugs at the ages and durations indicated. Medium was replaced every day, and embryos imaged as above at the time points indicated.

### Image analysis: Quantification of red blood cell speed

Time series datasets from all visible *x*-*y* plane vessels in the left optic tectum chosen were post processed in MATLAB. Using our custom codes (available on request), red blood cells (RBC) were segmented (by intensity based thresholding followed by pre-processing filters) in each frame followed by tracking of their centroids using an object-tracking algorithm.

RBC speed in *µm/s* was derived using the equation

(1) $$\mathrm{RB}C_{\mathrm{speed}}(\mu m/\text{s})=\mathrm{fps}\times \mathrm{scale}\times \sqrt{{\mathrm{Disp}}_{x}^{2}+{\mathrm{Disp}}_{y}^{2}}$$

where *fps* represents image acquisition rate (fps), *scale* is pixel to *µm* conversion (1 pixel = 0.609 *µm* × 0.609 *µm* with image dimension 487 *µm* × 365 *µm*), *Disp*_*x*_ and *Disp*_*y*_ are pixel displacements in ‘*x*’ and ‘*y*’ direction, respectively.

The speed obtained (from equation (1)) is then processed using modified interpolation and moving average filters (bin size of 3 s). Baseline, response and recovery speed are then compared in GraphPad Prism. For comparison between different groups, increase in speed is calculated as difference between average speed during response period (*t*_*resp*_) and during baseline period (*t*_*base*_) given by equation (2)

(2) $$\Delta \mathrm{RB}C_{\mathrm{speed}}=\frac{1}{t_{\mathrm{resp}}}\sum_{t_{\mathrm{resp}}}\mathrm{RB}C_{\mathrm{speed}}-\frac{1}{t_{\mathrm{base}}}\sum_{t_{\mathrm{base}}}\mathrm{RB}C_{\mathrm{speed}}$$

### Image analysis: Neuronal activation

Neuronal activation in the left optic tectum was quantified by GCaMP3 intensity (average over the entire left optic tectum) using Zen Black software (Zeiss). Relative change in fluorescence was calculated by equation (3)

(3) $$\frac{\Delta F}{F_{o}}=\frac{(F-B)-(F_{o}-B_{o})}{F_{o}-B_{o}}$$

where *F* is GCaMP3 intensity within an ROI which covers the whole left optic tectum, *B* is intensity of background area, *F*_*o*_ and *B*_*o*_ are baseline fluorescence (average of 10 s before stimulus onset) for optic tectum and background given by a general equation

(4) $$\bar{X}=\frac{1}{T}\sum_{t}X$$

where *X → F* for optic tectum and *X→B* for background area, *t* represents the integration time period averaged over total length, *T*, of the baseline. Time to peak and time to half peak were quantified for peaks induced by stimulus onset and stimulus offset.

### Image analysis: Vascular segmentation and quantification

Vascular features (branch points, vessel length, radius of vessels) were quantified using the Z stacks acquired as above. These were pre-processed and segmented using a processing pipeline similar to RBC segmentation. However, we used Otsu's thresholding^19^ instead of intensity-based thresholding to account for variable vessel fluorescence.

Morphological thinning^20^ was applied to obtain vascular centerlines (2D maximum intensity projections of the 3D stack), followed by branch points, vessel lengths, and radius quantification. For radius measurements, edge information was obtained from the binary segmented vasculature.^20^ Radius, *R*, was then calculated as Euclidean distance between the centerlines, *C*, and vasculature edges, *E*, for all the vessels using the following

(5) $$R=\sqrt{(C-E{)}^{2}}$$

To obtain distribution of radii, 2D radii for each vessel were used to calculate the frequency of each unique radius value in the left optic tectum.

### Image analysis: Quantification of claudin 5a intensity

*Tg(claudin5a:GFP;kdrl:mCherry)* was used to assess the BBB integrity, quantified using Z stacks acquired as above. Claudin 5a intensity was quantified as the average green channel intensity in masked out left optic tectum vessels (segmented using the vascular segmentation method described above). This was normalized to the vascular density in the left optic tectum for each animal in each group: mannitol or glucose; with or without SNP. This normalization was performed to reduce any bias due to treatment-induced differences in the vascular length.

### Experimental design and statistical analysis

Experiments were designed using the experimental design assistant of the NC3Rs online NC3RS-EDA tool.^21^ GraphPad Prism (La Jolla, CA®) was used for statistical comparisons. Before performing statistical analysis, data were tested for normality using the Shapiro–Wilk test. Neuronal calcium peaks and RBC speed in different time periods were compared using either a parametric one-way repeated measure ANOVA (RM-ANOVA, for normally distributed data) or a Friedman test (otherwise). Intergroup comparisons of neuronal activations and RBC speed for each time period (baseline, response and recovery) for various treatments (drugs/mannitol/glucose) were performed using parametric two-way ANOVA. For all ANOVA tests, intergroup comparisons were done using Sidak's multiple comparisons (post hoc) tests to obtain statistical difference between individual pairs of groups. Paired T-tests were used for two-group comparisons between tectal and non-tectal blood vessel RBC increases in response to light stimulation. Comparisons of vascular features such as number of tectal branch-points, vascular length and radii between two groups used unpaired *t*-tests or for more than two groups used parametric one-way ANOVA. *P* values < 0.05 were considered to be statistically significant. Data are mean ± standard deviation (s.d.), unless otherwise specified. Box plots represent the median with box edges representing 25% and 75% of the data and end lines indicating the maximum and minimum data values. Imaging was performed unblinded to treatment allocation; data analysis was automated and thus not subject to operator bias. Randomization of larvae to treatment group was not performed.

All data and codes used for the analysis are available on request.

## Results

### Zebrafish larvae develop neurovascular coupling between 6 and 8 dpf

We first examined whether zebrafish exhibit neurovascular coupling. We mounted non-anaesthetised eight days post fertilisation (dpf) transgenic zebrafish larvae in the chamber of a lightsheet microscope. Larvae expressed three transgenes: *Tg(kdrl:Has.HRAS-mCherry)*^14^ labelling endothelial cell membranes, *Tg*(*gata1:DsRed)*^15^ labelling erythrocytes allowing quantification of erythrocyte/RBC speed, and *Tg(nbt:GCaMP3)*^16^ which reports neuronal cytoplasmic calcium levels. An increase in GCamP3 fluorescence is indicative of an increase of neuronal electrical activity.^16,22^ Combining these allowed simultaneous quantification of neuronal activation, cerebrovascular anatomy, and RBC speed in individual cerebral vessels. After baseline imaging of the optic tectum, we exposed the animal to a pulse of red light for 8 s and continued imaging during and after this visual stimulus, with each stimulus protocol repeated twice per animal.

**Figure 1.**
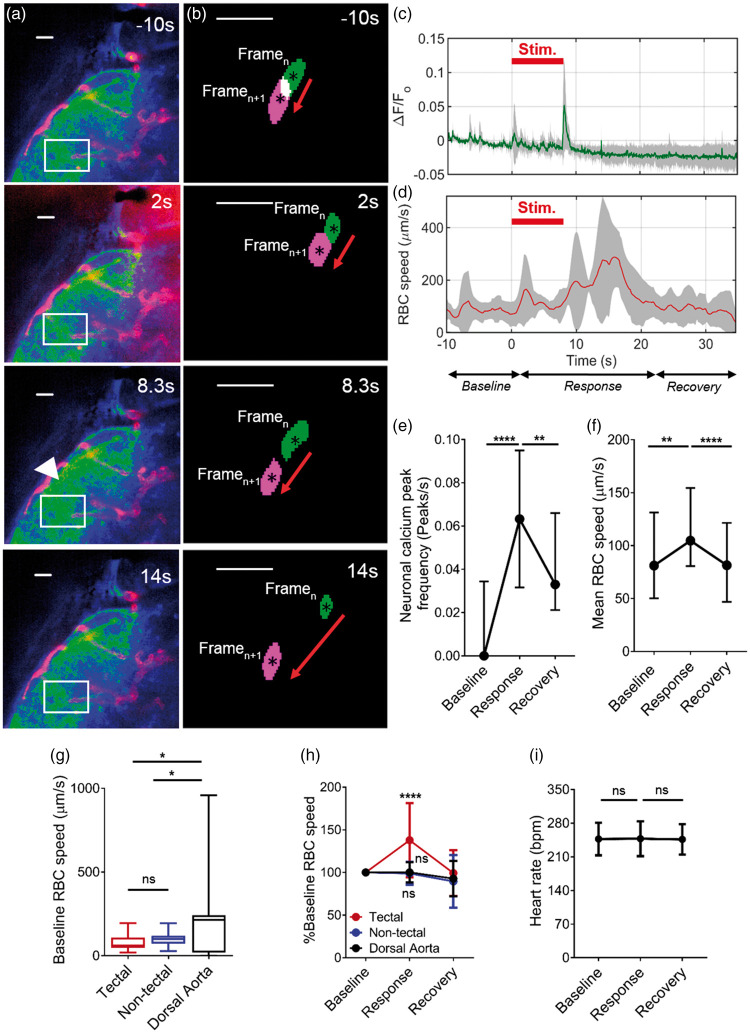
8 dpf zebrafish larvae display neurovascular coupling. (a) The left optic tectum of an 8 dpf *Tg(nbt:GCaMP3; kdrl:mCherry;gata1:DsRED)* embryo before (−10 s), during (2 s and 8.3 s) and after (14 s) visual stimulus by red light. Arrow indicates area of increased tectal calcium levels in response to the stimulus. (b) Segmented RBCs shown for two consecutive frames (Frame_n_ and Frame_n+1_) for corresponding time point of the neuronal responses shown in (a). Individual RBCs are labelled in green to represent Frame_n_ and magenta to represent Frame_n+1_. (c) Quantification of neuronal activation (*ΔF/F*_*o*_) in optic tectum over time (*n* = 5 larvae). Visual stimulus was administered 0–8 s (indicated on graph). Timeseries was divided into *baseline* (−10 – 0 s), *response* (0–20 s), and *recovery* (20–30 s) periods. (d) Erythrocyte (RBC) speed in tectal vessels in the same animals as (c). (e) Quantification of frequency of peaks in (*ΔF/F*_*o*_) as a measure of neuronal activation during baseline, response and recovery time periods (*n* = 40 larvae). (f) Mean RBC speed in the tectal vessels during baseline, response and recovery time periods in the same animals as (e). (g) Baseline RBC speed in tectal vessels, non-tectal (hindbrain and forebrain) vessels and dorsal aorta. (h) RBC speed expressed as percentage from the baseline for baseline, response and recovery for tectal vessels, non-tectal vessels and dorsal aorta (*n* = 17 larvae/group). (i) Beating heart rate (bpm) quantified for baseline, response and recovery time periods (*n* = 9 larvae/group). *p < 0.05, **p < 0.01, ****p < 0.0001. Scale bar represents 20 µm. Data in (e) and (f) are median and interquartile range (25% and 75% percentile). All other data are mean ± s.d.

Figure 1(a) shows representative images of the left optic tectum during these experiments. Neuronal GCamp3 (shown in green) increased in fluorescent intensity during the visual stimulus before returning to baseline 14 s after stimulus. This increase in fluorescence represents an increase in neuronal calcium levels, indicating increased neuronal activity. When we quantified this neuronal response (expressed as *ΔF/F*_*o*_) in five 8 dpf larvae (Figure 1(c)), we observed peaks in neuronal calcium levels. These peaks were observed at various times during the visual stimulus but most reliably seen in association with onset (9% of all animals) and offset (78% of all animals) of the visual stimulus. Representative examples of such responses are shown in Supplemental Figure 1(a) to (c).

We thus divided the duration of the experiment into three time periods relative to onset of visual stimulus: *baseline* (−10 to 0 s), *response* (0–20 s) and *recovery* (20–30 s).

We next examined RBC speed in the blood vessels of the left optic tectum to determine whether neuronal activation was associated with alteration in cerebral haemodynamics. Figure 1(b) shows the displacement of individual RBCs in the same animal as shown in Figure 1(a) at each timepoint, showing that this increased during stimulus. We quantified this response and found that RBC speed in the tectal vessels increased during the response, compared with baseline and recovery time periods (Figure 1(d)).

We quantified the neuronal and haemodynamic responses to visual stimulus in larger groups of animals. Frequency of Ca^2+^ peaks indicative of neuronal activation was significantly higher during the response compared with baseline and recovery periods, confirming that visual stimulus induced neural activation in the optic tectum (Figure 1(e)). We quantified the peak onset times (time to half peak amplitude (*t*_1/2_)^23^) for peaks induced by onset and offset of light stimulus; these were: onset 431 ± 254 ms and offset 366 ± 101 ms (mean ± s.d.), similar to values reported for GCaMP3 activation in previous studies.^23^

We next measured RBC speed in the tectal vessels to determine whether this altered in response to neuronal activation as evidence of neurovascular coupling. Mean RBC speed in the tectal blood vessels was significantly increased during the response compared with baseline and recovery periods (Figure 1(f)). We examined the temporal relationships between onset of visual stimulus, neuronal activation and change in RBC speed in each animal and found that the mean time between stimulus onset and peak neuronal activation was 6.9 ± 2.0 s (mean ± s.d.), and the mean time between stimulus onset and peak RBC speed was 13.3 ± 3.1 s (mean ± s.d.), (Supplemental Figure 1). To examine whether such an increase was due to a systemic effect, rather than localised to the area of neuronal activation, we measured RBC speed in both non-tectal cerebral blood vessels (in the hindbrain and forebrain) and the dorsal aorta in response to the same visual stimulus. As expected, baseline RBC speed was not different between tectal and non-tectal cerebral vessels, but was significantly higher in the dorsal aorta (Figure 1(g)). However, visual stimulus only increased RBC speed in the tectal vessels, not the non-tectal vessels or dorsal aorta (Figure 1(h)). To further exclude a systemic effect of the visual stimulus, we recorded the heart rate during baseline, response and recovery periods and found that this did not differ (Figure 1(i)).

To examine whether exposure to the 488 nm laser used to image the neuronal calcium reporter contributed to the alteration in RBC speed in the tectal vessels, we repeated experiments using an 8 s 488 nm laser illumination in place of visible red light while constantly recording RBC speed in the tectal vessels (via constant excitation with a 561 nm laser). Unlike the response to visible red light seen in Figure 1(e) and (f), the 488 nm laser excitation had no significant effect on RBC speed (Supplemental Figure 2).

To examine the effect of different stimulus protocols, we repeated these experiments using a 4 s and 12 s stimulus with red light (Supplemental Figure 3). We found that there was no significant difference in the change in neuronal peak frequency between baseline and stimulus periods with 4 s, 8 s, or 12 s visual stimulus, although there was a non-significant trend to a longer time to half peak in response to 4 s compared with 8 s and 12 s stimulus. However, the change in RBC speed in response to stimulus did not significantly differ between any of these stimulus durations.

We next examined the effect of four pulses of 4 s light (with 4 s intervals) to see whether this induced a more pronounced neurovascular response, but found that although this led to an increase in RBC speed earlier in the stimulus period, the neuronal and RBC speed responses were very similar to an 8 s stimulus (Supplemental Figure 4). We therefore used 8 s stimuli for all further studies here reported.

Since all animals underwent two stimulus trials, we examined the reproducibility and variability between these trials in each animal. We found no significant difference between either change in neuronal peak frequency or change in RBC speed between the first and second trial (Supplemental Figure 5).

Based on the data shown in Figure 1 and Supplemental Figures 1 to 5, we conclude that 8 dpf zebrafish embryos exhibit neurovascular coupling in the optic tectum in response to visual stimulus.

**Figure 2.**
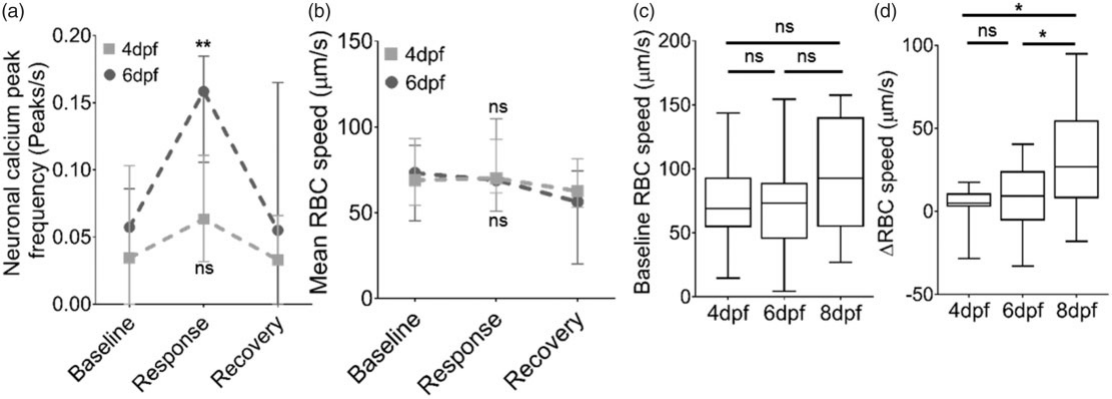
Neurovascular coupling in zebrafish develops after 6 dpf. (a) Quantification of frequency of peaks in (*ΔF/F*_*o*_) as a measure of neuronal activation during the baseline, response and recovery time periods for 4dpf and 6dpf (*n* = 17 larvae/group). (b) Mean RBC speed during baseline, response and recovery time periods in the same animals as (a) (*n* = 17 larvae/group). (c) Mean baseline RBC speed in tectal vessels in 4, 6 and 8 dpf zebrafish. (d) Change in RBC speed (ΔRBC) in response to stimulus in 4, 6, and 8 dpf (*n* = 17 larvae/group). *p < 0.05, **p < 0.01. Data in (a) and (b) are mean ± s.d.

Rats develop classical neurovascular coupling around p23.^24^ We therefore examined whether a similar developmental threshold of neurovascular coupling exists in zebrafish. Visual stimulus had no effect on neuronal activation (frequency of calcium peaks) in 4 dpf embryos (Figure 2(a)) preventing assessment of whether neurovascular coupling exists at this age. In comparison, 6 dpf larvae exposed to visual stimulus did show a significant increase in neuronal activation in the optic tectum (Figure 2(a)), of a similar magnitude to that seen in 8 dpf larvae in Figure 1(e). However, the increase in neural activation induced by visual stimulus in 6 dpf larvae was not accompanied by an increase in RBC speed (Figure 2(b)) as seen in 8 dpf larvae. This divergent haemodynamic response between different developmental stages was not due to different baseline tectal vessel RBC speed (Figure 2(c)). Therefore, 8 dpf, but not 6 dpf zebrafish larvae exhibited an increase in RBC speed (ΔRBC speed) in response to visual stimulus-evoked neural activation (Figure 2(d)). Thus, our data demonstrate that, similarly to mammals, zebrafish exhibit a developmental threshold for neurovascular coupling, which occurs between 6 and 8 dpf.

### The effect of glucose exposure on zebrafish cerebrovascular anatomy and neurovascular coupling

**Figure 3.**
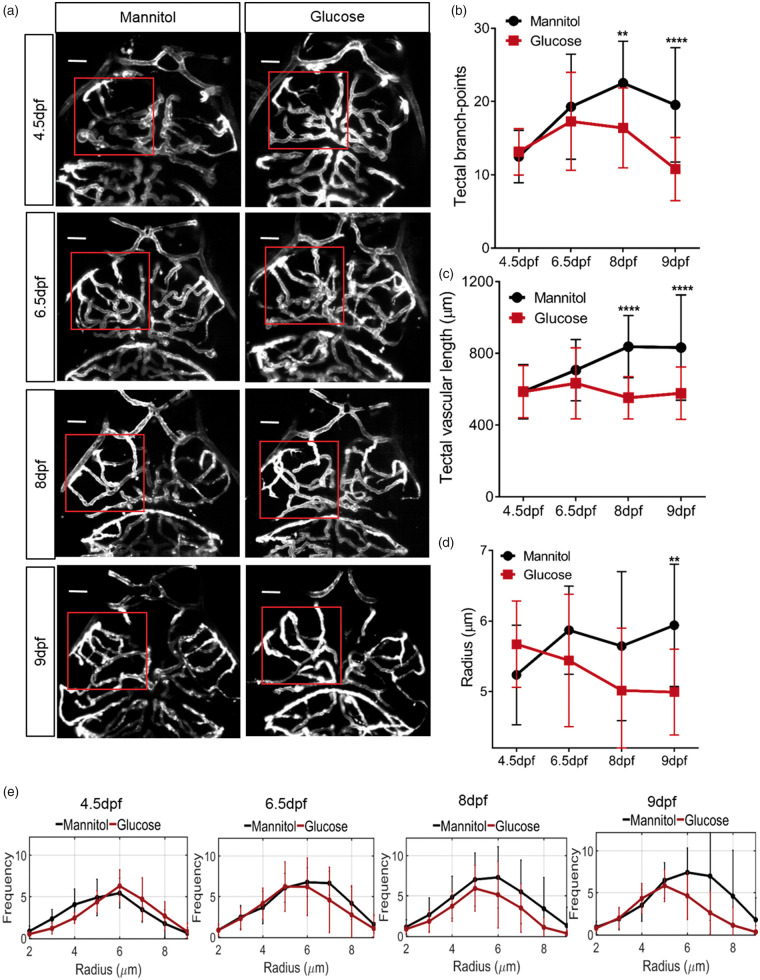
Effect of glucose exposure on cerebrovascular patterning in zebrafish. (a) Representative micrographs of cerebral vessels exposed to 20 mM mannitol or glucose from 4 dpf for 12 h, 60 h, 96 h and 120 h. Square indicates region of left optic tectum quantified in (b)–(e). (b) Number of tectal vessel branchpoints (*n* = 17, 16, 15 and 20 larvae for 12 h, 60 h, 96 h and 120 h exposure, respectively). (c) Total tectal vessel length in same animals as (b). (d) Mean tectal vessel radius in same animals as in (b). (e) Histograms of tectal vessel radii of tectal vessels in same animals as in (b). Scale bar represents 20 µm. Data are mean ± s.d.

**Figure 4.**
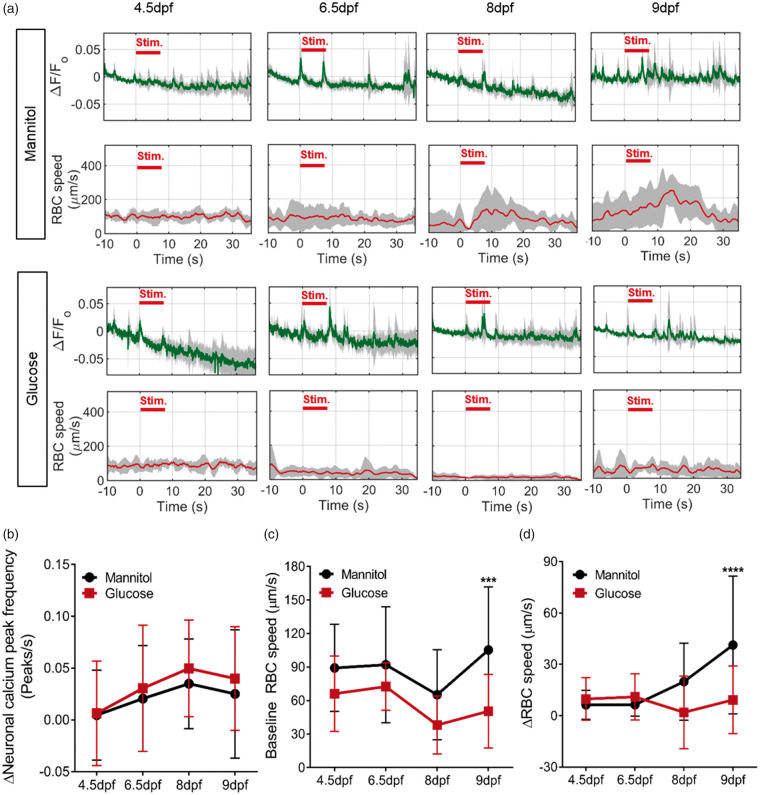
Effect of glucose exposure on neurovascular function in zebrafish. (a) Time series of neuronal activation (*ΔF/F*_*o*_) and tectal vessel RBC speed in zebrafish exposed to 20 mM mannitol or glucose from 4 dpf for 12 h, 60 h, 96 h and 120 h (*n* = 5 larvae/group). (b) Change in neuronal calcium peak frequency in the left optic tectum during response compared to baseline periods (*n* = 17, 17, 15 and 20 for 12 h, 60 h, 96 h and 120 h exposure, respectively). (c) Baseline RBC speed at the same time points in (b) and (c). (d) Change in RBC speed (ΔRBC) between baseline and response time periods for the same animals as (b). ***p < 0.005, ****p < 0.0001. Data are mean ± s.d.

We next examined the effect of hyperglycemia induced by incubation in glucose on cerebrovascular anatomy and function in zebrafish. Larvae were incubated in 20 mM glucose (a level seen in poorly controlled diabetes) or 20 mM mannitol (osmotic control) at 4 dpf for 12, 60, 96 and 120 h to examine the effect of glucose on cerebrovascular patterning (Figure 3) and neural activation and tectal vessel RBC speed in response to visual stimulus (Figure 4).

Figure 3(a) shows representative micrographs of the cerebral vessels of larvae exposed to glucose or mannitol. Glucose exposure for 12 h (from 4 to 4.5 dpf) or 60 h (from 4 to 6.5 dpf) had no significant effect on tectal vessel anatomy. However, 96 h (from 4 to 8 dpf) glucose exposure significantly impaired cerebrovascular patterning judged by branchpoint number (Figure 3(b)), and total tectal vessel length (Figure 3(c)) though mean tectal vessel radius was not significantly different (Figure 3(d) and (e)). By 120 h (from 4 to 9 dpf) exposure, all these measures of vascular patterning were significantly impaired by glucose (Figure 3(b) to (e)).

When we examined the effect of glucose on the neural response to visual stimulus, we found no significant difference between glucose and mannitol exposed larvae up to 120 h exposure (4 to 9 dpf, Figure 4(a) and (b)). Baseline RBC speed (prior to visual stimulus) was not significantly different at 4.5 dpf, 6.5 dpf or 8 dpf in the glucose exposed group, but was significantly reduced at 9 dpf (120 h of glucose exposure) (Figure 4(c)).

Consistent with the observation that neurovascular coupling does not develop in zebrafish until after 6 dpf, ΔRBC speed in response to neuronal activation was low and not significantly different between glucose and mannitol-treated animals at 4.5 and 6.5 dpf (12 h and 60 h treatment) (Figure 4(d)). However, although 8 dpf larvae treated to mannitol displayed a significant increase in tectal vessel RBC speed in response to neural stimulus, glucose-treated embryos did not. By 9 dpf (120 h after commencing treatment), ΔRBC speed in the optic tectum in response to visual stimulus was significantly reduced by glucose compared with mannitol (Figure 4(d)). This result cannot be explained by an absence of glucose in the mannitol-treated group as repeating these experiments found no difference between animals treated for five days from 4 dpf with 20 mM mannitol compared with animals treated with 15 mM mannitol plus 5 mM glucose (Supplemental Figure 6).

Collectively, our data show that chronic exposure to 20 mM glucose (but not 5 mM) impairs cerebrovascular anatomy, baseline tectal vessel RBC speed, and neurovascular coupling in zebrafish.

### The NO donor SNP rescues the adverse effects of glucose on cerebrovascular anatomy and neurovascular coupling in zebrafish

**Figure 5.**
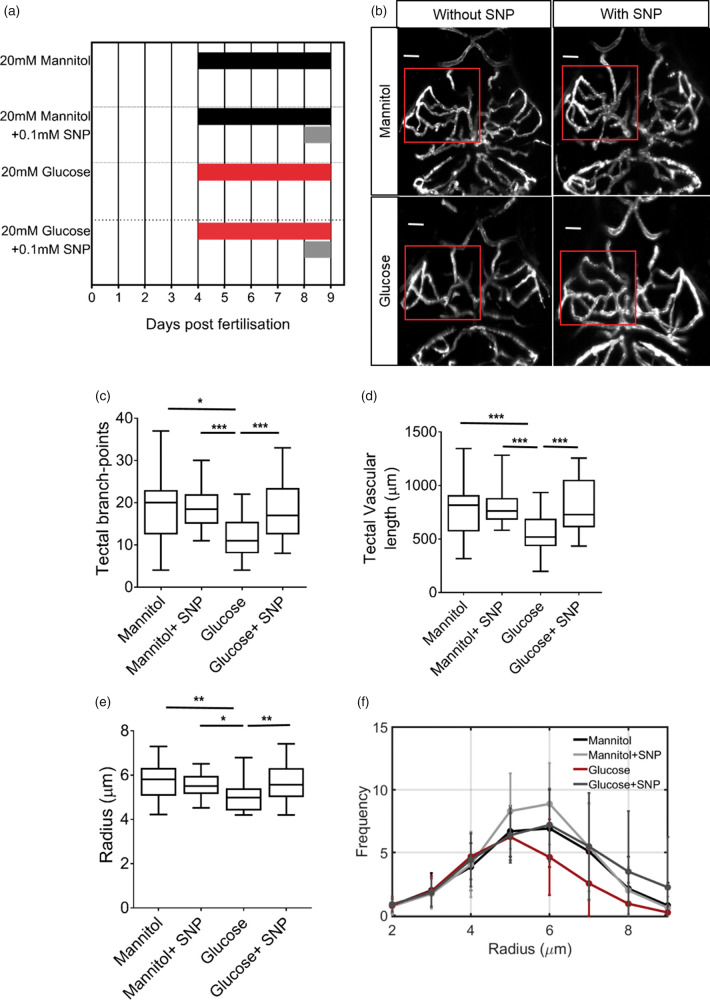
Sodium nitroprusside reverses the effect of glucose exposure on cerebrovascular patterning. (a) Gannt chart indicating treatment groups and duration of drugs exposures for experiments in Figures 6 and 7. (b) Representative micrographs of cerebral vessels of 9 dpf *Tg(kdrl:mCherry)* larvae exposed to mannitol or glucose ± SNP. (c) Quantification of number of tectal vascular branch points in all groups (*n* = 23 larvae/group). (d) Quantification of total tectal vessel length in same animals as in (c). (e) Mean tectal vessel radius for the same animals as in (c) (*n* = 23 larvae/group). (f) Histogram of radii of vessels in the left optic tectum in the same animals as in (c). Scale bar represents 20 µm. *p < 0.05, **p < 0.01, ***p < 0.005. Scale bar represents 20 µm. Data in (f) are mean ± s.d.

Multiple reports show that nitric oxide (NO) generation is impaired in diabetes.^25–27^ Since both vascular development and neurovascular coupling are NO-dependent, we examined whether the NO donor SNP could ameliorate the negative effects of exposure to glucose. We therefore exposed 4 dpf embryos to 20 mM glucose or mannitol for 120 h until 9 dpf (as in Figures 3 and 4) and examined the effect of co-treatment with or without 0.1 mM SNP for 24 h starting at 8 dpf (96 h after starting glucose or mannitol exposure. Figure 5(a) summarises the timings and durations of drug treatment in these four groups). Five days exposure to mannitol plus 24 h treatment with SNP had no effect on tectal branchpoint number, total vascular length, or mean vessel radii compared with mannitol alone (Figure 5(b) to (f)). As we previously found, 20 mM glucose from 4 to 9 dpf reduced tectal vessel branching, length, and mean radius (Figure 5(b) to (f)). However, 24 h SNP co-treatment (started at 8 dpf) completely reversed the effects of glucose on cerebrovascular anatomy (Figure 5(b) to (f)).

**Figure 6.**
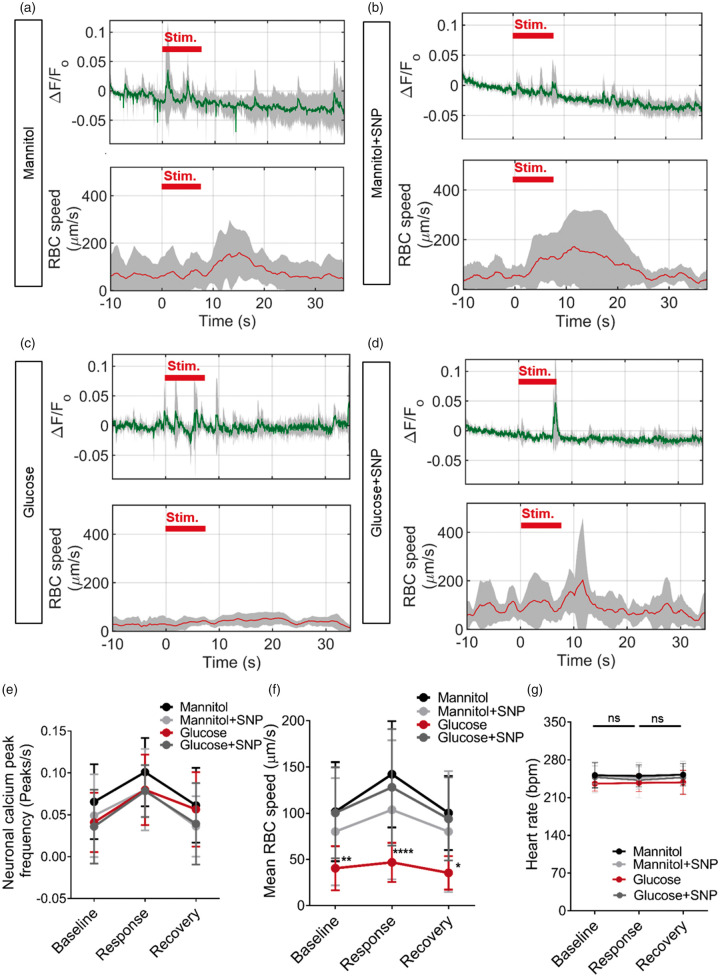
Sodium nitroprusside reverses the effect of glucose on neurovascular coupling. (a)–(d) Time series of neuronal activation (*ΔF/F*_*o*_) and tectal vessel RBC speed in zebrafish (5 larvae/group) exposed to: (a) 20 mM mannitol for 120 h from 4 to 9 dpf. (b) 20 mM mannitol for 120 h from 4 to 9 dpf and 0.1 mM SNP for 24 h from 8 to 9 dpf. (c) 20 mM glucose for 120 h from 4 to 9 dpf. (d) 20 mM glucose for 120 h from 4 to 9 dpf and 0.1 mM SNP for 24 h from 8 to 9 dpf. (e) Frequency of peaks of neuronal activation during baseline, response and recovery time periods in mannitol or glucose exposed larvae with or without co-treatment with 0.1 mM SNP (*n* = 20 larvae/group). (f) RBC speed for baseline, response and recovery for same animals in (e). (g) Beating heart rate (bpm) quantified for baseline, response and recovery time periods for larvae exposed to 20 mM mannitol, 20 mM glucose with or without SNP treatment. *p < 0.05, **p < 0.01, ***p < 0.001, ****p < 0.0001. Data are mean ± s.d.

We next examined the effect of 24 h SNP co-treatment on neurovascular coupling in the same animals as shown in Figure 5. SNP co-treatment with mannitol did not affect neurovascular coupling compared with mannitol alone (Figure 6). As we observed earlier, glucose exposure from 4 to 9 dpf significantly impaired both baseline tectal vessel RBC speed and the visual stimulus-evoked increase in RBC speed, while having no effect on the neuronal calcium response to a visual stimulus; 24 h co-treatment with SNP prevented this impairment in both baseline RBC speed and visual stimulus-induced increase in RBC speed (ΔRBC speed, Figure 6(f)). These differences were not due to any difference in heart rate as this did not differ between baseline, response or recovery periods, or between treatment groups (Figure 6(g)). Thus, 24 h treatment with SNP both prevented the cerebrovascular patterning defects associated with 120 h glucose exposure, and furthermore prevented glucose-induced impairment of neurovascular coupling.

**Figure 7.**
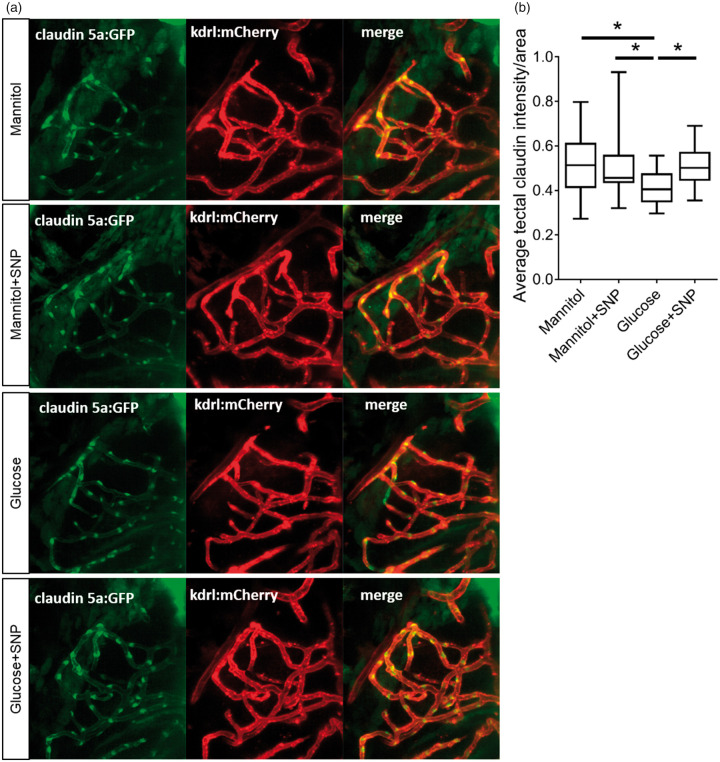
*Claudin 5a:GFP* expression in tectal vessels in zebrafish larvae treatment with mannitol or glucose with or without SNP. (a) Representative micrographs of *Tg(claudin5a:GFP;kdrl:mCherry)* exposed to 20 mM mannitol for five days or 20 mM glucose for five days with or without SNP (one day treatment) imaged at 9 dpf. (b) Quantification of mean GFP fluorescence in the tectal vessels (*n* = 23, 19, 24 and 19 larvae for mannitol, mannitol+SNP, glucose and glucose+SNP-treated larvae, respectively).

We next sought to examine whether the effects of glucose in our model might be mediated via alteration of the blood–brain barrier (BBB). Claudin 5 is a key component of the BBB,^28^ and a transgenic zebrafish has recently been generated that expresses GFP driven by the *claudin5a* promoter.^17^ We therefore examined the effect of five days mannitol or glucose exposure from 4 dpf, with or without SNP for one day from 8 dpf (as shown in Figure 5(a)), on expression of the *claudin5a:GFP* reporter in the tectal blood vessels. Figure 7(a) shows representative micrographs from these animals, revealing clear expression of GFP in the tectal vessels. When we quantified GFP expression in these vessels, this was significantly reduced by glucose exposure, which was rescued by SNP co-treatment (Figure 7(b)). These data suggest that the negative effects of glucose on cerebrovascular development and neurovascular coupling may be mediated, at least in part, by alteration of BBB structure or integrity.

## Discussion

In this paper, we establish a novel approach for investigating neurovascular coupling, exploiting powerful imaging and image analysis techniques in sophisticated compound transgenic zebrafish that allow totally non-invasive simultaneous assessment of multiple aspects of cerebrovascular anatomy and cellular function and physiology. We prove the utility of this model by showing that exposure to elevated glucose levels as seen in diabetes impairs both neurovascular coupling and cerebrovascular patterning in zebrafish, and that these deficits are reversed or prevented by the NO donor SNP.

The zebrafish is well established as a model of embryonic development, whose advantages are increasingly applied to the study of physiology and disease-related mechanisms. We describe the first non-mammalian example of neurovascular coupling, finding that zebrafish exhibit a response to neural stimulus analogous to rodents and humans. This suggests neurovascular coupling is evolutionarily conserved, presumably predating the phylogenetic divergence of mammals from teleosts. Our zebrafish model is less expensive and time-consuming than rodent models and requires no invasive surgery. It could be used to address a range of questions that would be challenging to test in rodents, such as rapid evaluation of the effect of multiple small molecule drugs.^29^ The ease of genetic manipulation of zebrafish,^30^ the availability of thousands of zebrafish mutants,^31,32^ and the speed at which candidate genes can be targeted by CRISPR/Cas9 mutagenesis,^33^ would allow large genetic screens to identify the molecular contributors to neurovascular function.

Although our data clearly demonstrate that zebrafish exhibit neurovascular coupling, it is not known whether the mechanisms of this are entirely conserved between zebrafish and mammals; 70% of human disease-related genes have zebrafish orthologues,^34^ and it is likely that the same broad mechanisms control zebrafish neurovascular coupling, but it remains to be determined whether all known or potential regulators of mammalian neurovascular coupling exist in zebrafish.

Diabetes is a complex disease, of which elevated blood glucose, although an essential feature, is only one facet. Type II diabetes is associated with both hyperinsulinaema and insulin resistance, while type I diabetes is associated with low insulin production, all of which may have effects separate to elevated glucose. Exposure to glucose is therefore not a model of diabetes per se, but the ability to examine the effect of this single (and defining) feature of diabetes has utility. Other studies have applied this approach,^35–37^ although often using far higher levels of glucose than seen in human diabetics. The average blood glucose level in adult zebrafish is around 3.1–7.1 mM (56–130 mg/dL^37^), similar to a non-diabetic human. We therefore examined the effect of exposure to glucose concentrations often found in diabetic humans (20 mM/360 mg/dL). Adult *pdx-1* mutant zebrafish which develop diabetes have mean blood glucose levels of 219 mg/dL (12 mM), while streptozotocin-induced pancreatic damage raises blood glucose levels in adult zebrafish to similar levels to diabetic humans.^38^ Although no study has successfully measured blood glucose in zebrafish larvae or embryos, these data suggest our study examined the effect of a relevant level of glucose exposure. The negative effects of glucose were not apparent after 60 h exposure, but required 120 h to manifest and became more marked with longer exposure, which is in keeping with the chronic nature of diabetic complications. Hyperglycemia is often present in type II diabetics for years prior to and after diagnosis. Even subtle effects of hyperglycemia on cerebrovascular function might therefore accumulate to eventually induce clinical sequelae.

Gestational diabetes complicates 1–14% of human pregnancies and exposes a non-diabetic human fetus to elevated glucose which crosses the placenta from the diabetic mother.^39^ It is increasingly apparent that this exposure increases the risk of cardiovascular disease in the offspring's later life.^40,41^ Our data strongly suggest that sustained exposure to hyperglycemia during embryogenesis compromises fetal cerebrovascular development and function. Although not conclusive, evidence exists to suggest that exposure to hyperglycaemia in utero may negatively affect postnatal cognition in humans.^42^ This underlines the importance of understanding the effect of glucose on the developing embryonic brain, as in our study.

Diabetes is associated with dysfunctional neurovascular coupling in both humans^43^ and rodent models.^6^ In addition, diabetes impairs angiogenesis and vascular remodelling in both humans and mice,^44,45^ although the effect on cerebrovascular development has been less well studied. In our model, chronic exposure to elevated glucose levels results in cerebrovascular patterning defects and impairs neurovascular coupling in 9 dpf zebrafish. Our findings extend those in adult zebrafish which previously demonstrated diabetes-associated micro and macrovascular disease.^46,47^

Since diabetes and hyperglycemia are associated with impaired endothelial NO generation^25,48^ and both angiogenesis^49^ and neurovascular coupling^50^ are NO dependent, we speculated that the observed defects arise as a result of reduced bioavailability of endothelial NO. Hence, we tested the effect of an NO donor, SNP. Administration of SNP resulted in a marked reversal of vascular patterning and neurovascular coupling defects. These results suggest that diabetes-associated neurovascular and cerebrovascular defects may be treatable by NO donating drugs which are already widely used clinically to treat cardiovascular diseases such as hypertension and angina pectoris.^13,51^

The exact mechanisms underlying the effects of glucose, and the ability of SNP to rescue these in our model remain to be elucidated. We found that a *claudin5a* transgene expression was reduced by glucose and rescued by SNP co-treatment in a manner which mirrors the effects of these treatments on cerebrovascular patterning and neurovascular coupling. This interesting observation might support the possibility that the effects of glucose are mediated by alteration of the structure of the blood–brain barrier. It is also possible that increasing NO reverses high glucose-evoked dysfunctional endothelial-dependent vasodilation^52,53^ and reduced NO-mediated vasodilation.^54,55^ However, in diabetic rats, deficits in retinal functional hyperaemia were reversed by inhibition of inducible NO synthase (iNOS^56^). Therefore, future work should elucidate the mechanisms underlying high glucose-evoked neurovascular and cerebrovascular deficits in zebrafish and their improvement by the addition of NO. Although the safety of any drug in pregnancy is uncertain, NO donors are one of the few classes of agents that are not prohibited during pregnancy and can be used where justified by the clinical benefits (FDA class C). A clinical study to examine the effect of NO donors to prevent hyperglycemia-induced impairment of fetal cerebrovascular development is therefore not inconceivable, although would require a better understanding of the lifetime risk to the fetus of gestational diabetes. Since this risk is challenging to quantify in humans without large longitudinal cohort studies, models such as ours contribute to understanding the potential magnitude of any effect, and may help in identifying markers (such as impaired neurovascular coupling, which can be measured non-invasively by fMRI in humans) to examine in such studies.

Cognitive decline in neurodegenerative diseases^4,57,58^ is linked to neurovascular dysfunction and vascular defects seen in diabetes.^59,60^ Further work is thus needed to assess whether improvements in neurovascular coupling due to NO donor administration provide an effective means of ameliorating diabetes-associated cognitive dysfunction.^61^

## Supplemental Material

Supplemental material for The effect of hyperglycemia on neurovascular coupling and cerebrovascular patterning in zebrafish Click here for additional data file. Supplemental material for The effect of hyperglycemia on neurovascular coupling and cerebrovascular patterning in zebrafish by Karishma Chhabria, Karen Plant, Oliver Bandmann, Robert N Wilkinson, Chris Martin, Elisabeth Kugler, Paul A Armitage, Paola LM Santoscoy, Vincent T Cunliffe, Jan Huisken, Alexander McGown, Tennore Ramesh, Tim JA Chico and Clare Howarth in Journal of Cerebral Blood Flow & Metabolism
