# Supplementary material for: The effect of hyperglycemia on neurovascular coupling and cerebrovascular patterning in zebrafish
Source: J Cereb Blood Flow Metab. 2018 Nov 6;40(2):298–313. doi: 10.1177/0271678X18810615 (PMC6985997; doi:10.1177/0271678X18810615)
Supplement: Supplemental material for The effect of hyperglycemia on neurovascular coupling and cerebrovascular patterning in zebrafish [file Supplemental_material16.pdf]

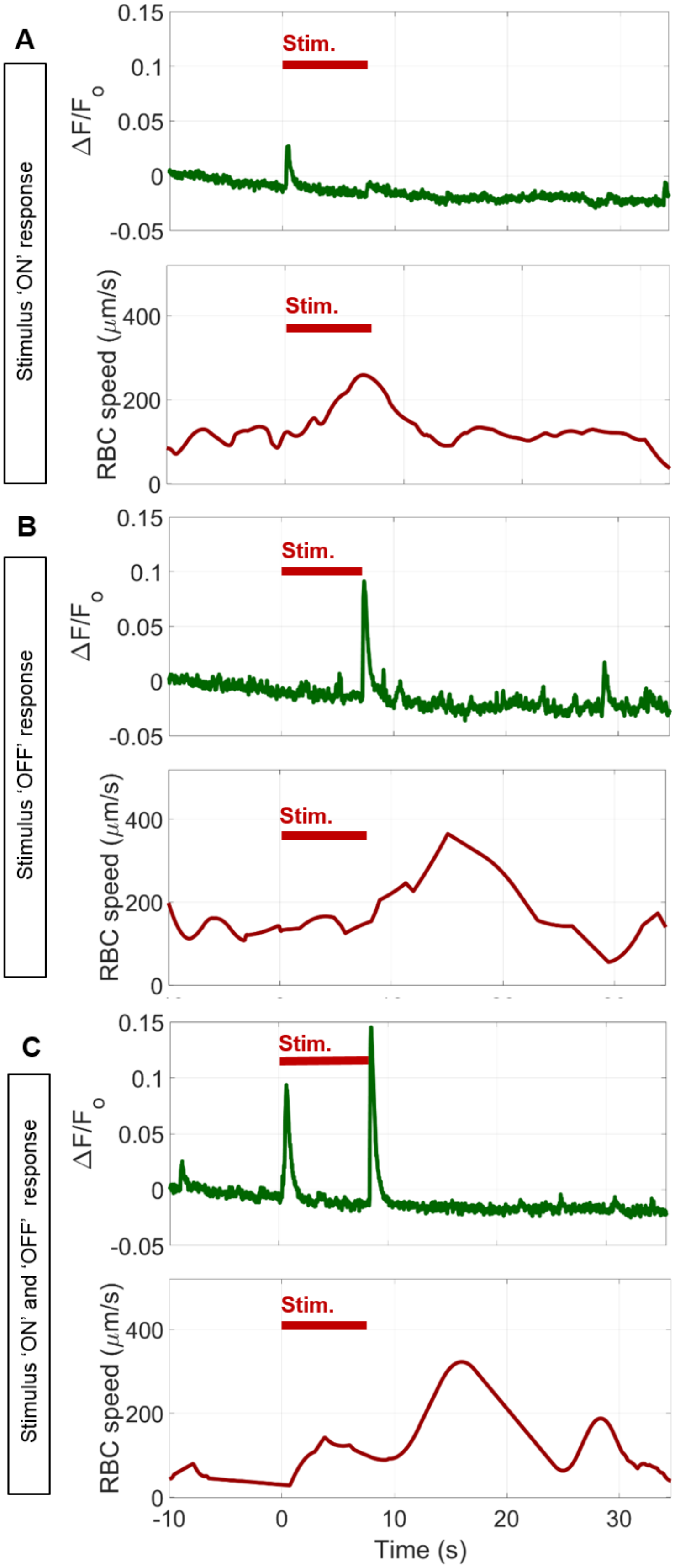

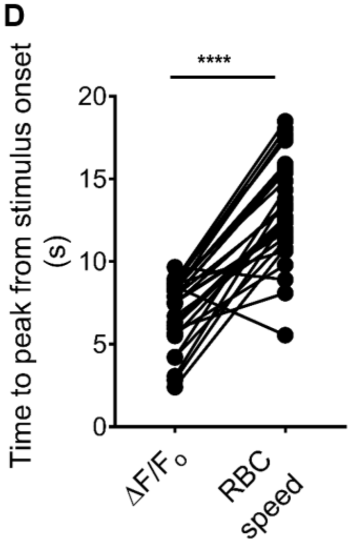

Supplemental Figure 1: Temporal relationships between onset of visual stimulus, neuronal activation and change in RBC speed at 8dpf. **A-C**: Quantification of neuronal activation (*ΔF/Fo*) in the optic tectum and RBC speed in tectal vessels over time for example animals in which neuronal calcium peaks were associated with light stimulus onset **(A)**,offset **(B)** or both onset and offset **(C)**. Visual stimulus is administered 0-8s (indicated on the graphs by red bar). **D**: Quantification of mean time to peak for neuronal calcium responses (*ΔF/Fo*) and RBC speed with respect to stimulus onset (n= 30 larvae).

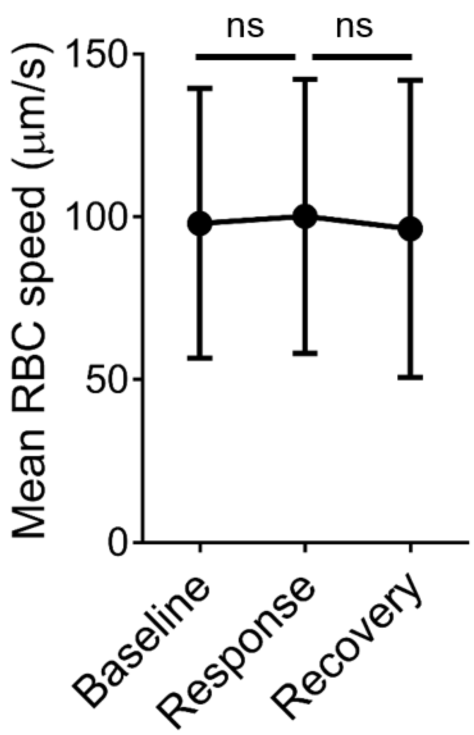

Supplemental Figure 2: RBC speed does not change in response to 488nm as stimulus light. RBC velocity quantification in tectal vessels of 8dpf larvae with 488nm imaging laser as the stimulus (8s). Data are mean ± s.d.

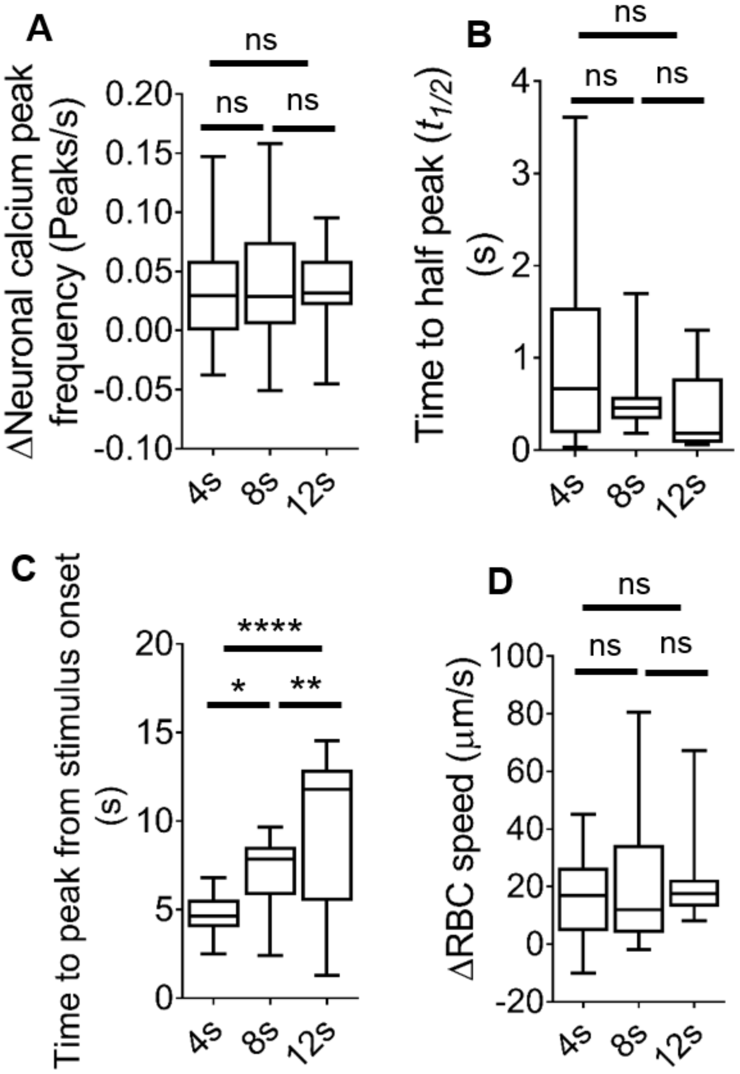
A
ΔNeuronal calcium peak frequency (Peaks/s)
0.20
0.15
0.10
0.05
0.00
-0.05
-0.10
ns
ns
ns
4s
8s
12s
B
Time to half peak ($t_{1/2}$) (s)
4
3
2
1
0
ns
ns
ns
4s
8s
12s
C
Time to peak from stimulus onset (s)
20
15
10
5
0
****
*
**
4s
8s
12s
D
ΔRBC speed (μm/s)
100
80
60
40
20
0
-20
ns
ns
ns
4s
8s
12s

[redacted]

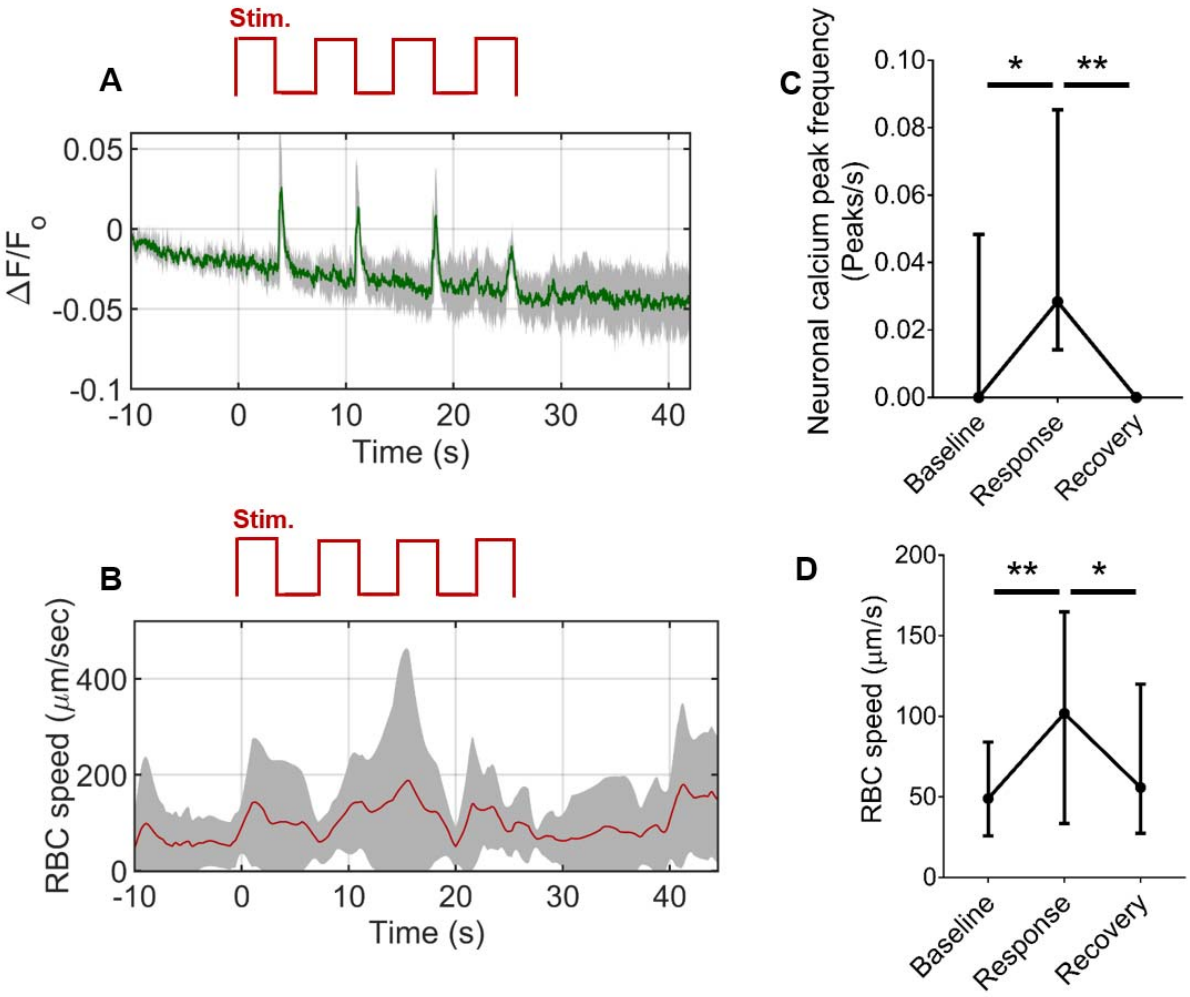

Supplemental Figure 4: Neurovascular coupling in response to pulsed light stimulus. A: Quantification of neuronal activation (*ΔF/Fo*) in optic tectum in response to four 4 second pulses (red trace) for five larvae at 8dpf. **B:** Quantification of tectal vessel RBC speed in response to the pulse stimulus for the same animals as in **A**. **C**: Quantification of calcium peak frequency for baseline, response and recovery for 8dpf larvae in response to four 4 second pulsed stimulus (n=14 larvae). **D:** Quantification of mean RBC speed for baseline, response and recovery for the same animals as in **C** (n=14 larvae). Data in **A** and **B** are mean ± s.d. Data in **C** and **D** are median and interquartile range (25% and 75% percentile).

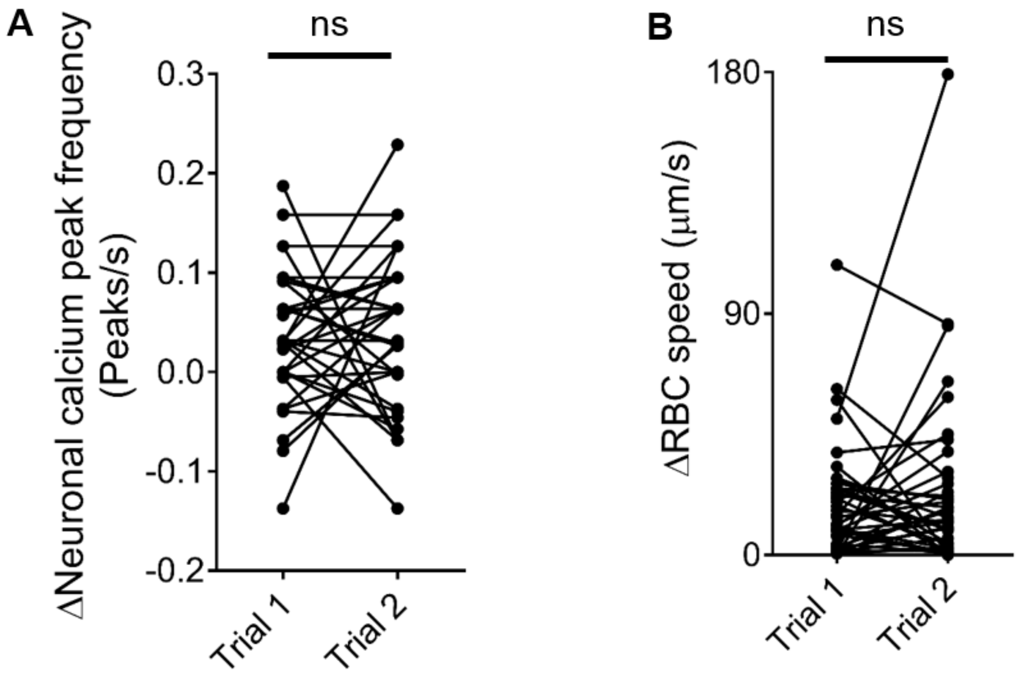

Supplemental Figure 5: Trial to trial variability A: Comparison of change in neuronal calcium peak frequency during response time period compared to baseline for trial 1 and trial 2 (n=40 larvae) at 8dpf. **B:** Comparison of change in RBC speed during response time period compared to baseline for trial 1 and trial 2 for the same animals as in **A**.

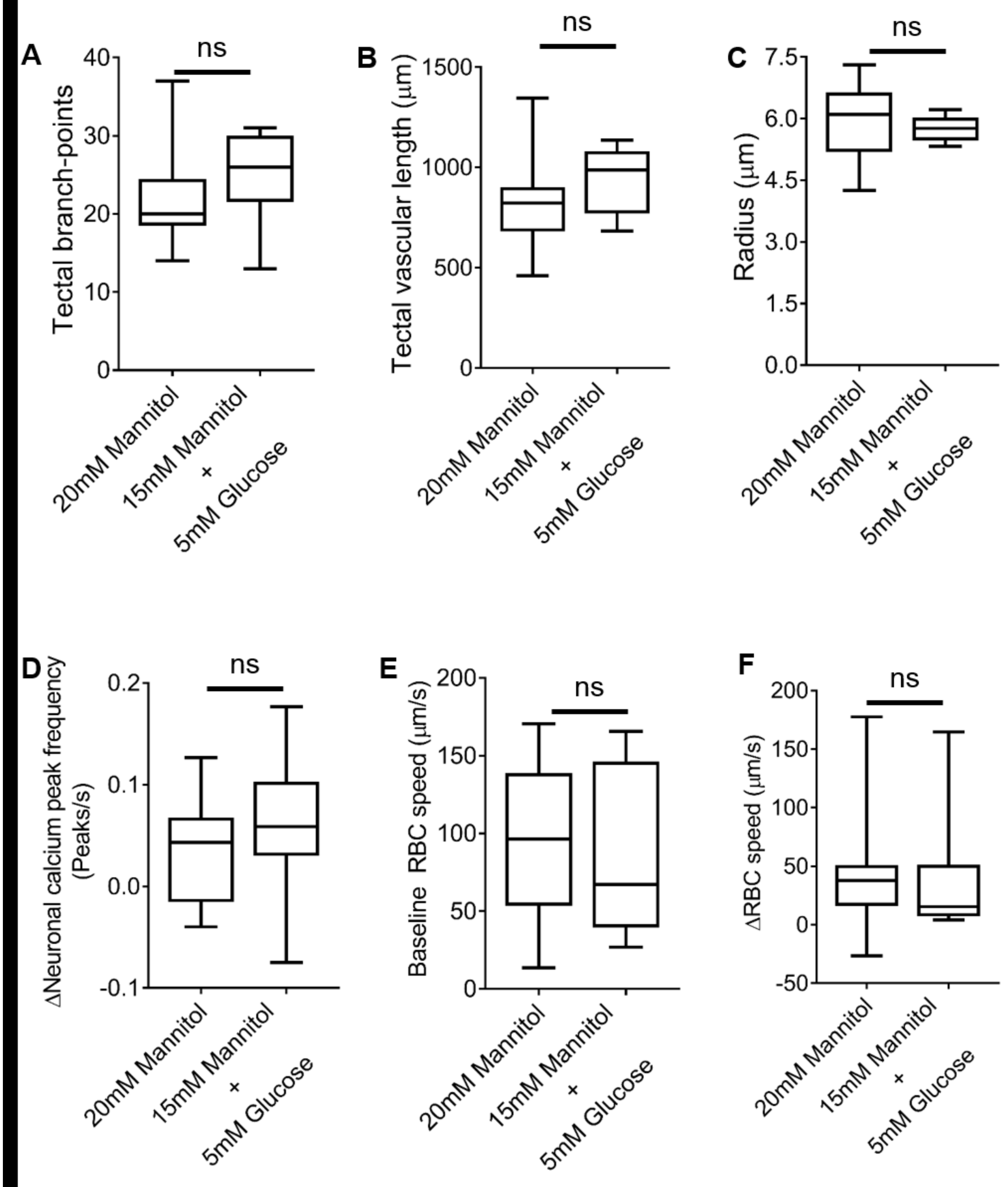

Supplemental Figure 6: Cerebrovascular patterning and neurovascular coupling in larvae exposed to 20mM mannitol and 15mM mannitol + 5mM glucose: A-C: Comparison of cerebrovascular patterning (number of tectal branch-points **(A)**, tectal vascular length **(B)** and tectal vessel radius **(C)**) in larvae exposed to 5d of 20mM mannitol or 15mM mannitol + 5mM glucose (n=15 larvae/group) imaged at 9dpf. **D-F:** Comparison of change in neuronal calcium peak frequency **(D)**, baseline RBC speed **(E)** and *ΔRBC* speed **(F)** in response to visual stimulus in the same animals as in **A-C** (n=15 larvae/group)
